# Perturbation of TG‐43 parameters of the brachytherapy sources under insufficient scattering materials

**DOI:** 10.1120/jacmp.v14i3.4228

**Published:** 2013-05-06

**Authors:** Mehdi Zehtabian, Sedigheh Sina, Reza Faghihi, Ali Meigooni

**Affiliations:** ^1^ Department of Medical Engineering School of Mechanical Engineering Shiraz Fars Iran; ^2^ Radiation Research Center Shiraz University Shiraz Fars Iran; ^3^ Comprehensive Cancer Centers of Nevada Las Vegas Nevada USA

**Keywords:** TG‐43 brachytherapy, dosimetry, Monte Carlo, missing tissue, overlaying tissue

## Abstract

In the recommendations of Task Group #43 from American Association of Physicists in Medicine (AAPM TG43), methods of brachytherapy source dosimetry are recommended, under full scattering conditions. However, in actual brachytherapy procedures, sources may not be surrounded by full scattering tissue in all directions. Clinical examples include high‐dose‐rate (HDR) brachytherapy of the breast or low‐dose‐rate (LDR) brachytherapy of ocular melanoma using eye plaque treatment with ${\;}^{125}I$ and ${\;}^{103}Pd$. In this work, the impact of the missing tissue on the TG‐43–recommended dosimetric parameters of different brachytherapy sources was investigated. The impact of missing tissue on the TG‐43–recommended dosimetric parameters of ${\;}^{137}Cs,{\;}^{192}Ir$, and ${\;}^{103}Pd$ brachytherapy sources was investigated using the MCNP5 Monte Carlo code. These evaluations were performed by placing the sources at different locations inside a $30\times 30\times 30\;{cm}^{3}$ cubical water phantom and comparing the results with the values of the source located at the center of the phantom, which is in a full scattering condition. The differences between the thickness of the overlying tissues for different source positions and the thickness of the overlying tissue in full scattering condition is referred to as missing tissue. The results of these investigations indicate that values of the radial dose function and 2D anisotropy function vary as a function of the thickness of missing tissue, only in the direction of the missing tissue. These changes for radial dose function were up to 5%, 11%, and 8% for ${\;}^{137}Cs,{\;}^{192}Ir$, and ${\;}^{103}Pd$, respectively. No significant changes are observed for the values of the dose rate constants. In this project, we have demonstrated that the TG‐43 dosimetric parameters may only change in the directions of the missing tissue. These results are more practical than the published data by different investigators in which a symmetric effect of the missing tissue on the dosimetric parameters of brachytherapy source are being considered, regardless of the implant geometry in real clinical cases.

PACS number: 87.53.JW

## I. INTRODUCTION

In 1995, Task Group 43 (TG‐43) of the American Association of Physicists in Medicine (AAPM) introduced a worldwide recommendation for dosimetry of brachytherapy sources.^(1)^ The updated version of TG‐43 formalism (known as TG‐43U1) was published in 2004^(2)^ to eliminate shortcomings in the original formalism, to clarify definitions such as the active lengths of the sources with different geometry, and also to introduce the dosimetry of the sources that were not available during the preparation of the original report. The original and updated TG‐43 formalism clearly defines the clinically required quantities for various low‐energy brachytherapy sources, such as air kerma strength, dose rate constant, radial dose function, 2D and 1D anisotropy functions, and geometric function in a homogeneous water phantom.^1^, ^2^ Since 1995, many investigations explored the differences of the brachytherapy source dosimetry in heterogeneous phantoms as compared to the homogenous water phantom.^3^, ^4^ In addition, the influence of phantom size and shape on TG‐43 dosimetric parameters of the brachytherapy sources have been evaluated by several investigators.^5^, ^6^, ^7^, ^8^, ^9^, ^10^, ^11^ It is important to note that the use of Monte Carlo simulation was very significant by providing dosimetric information for brachytherapy source with high precision for these investigations.^12^, ^13^, ^14^ The results indicated that phantom size had affected the radial dose function, g(r), but there was no significant effect on the anisotropy function, F(r, θ). It should be noted that all of these investigations were based on the assumptions that the brachytherapy source was placed at the center of a phantom of varying size, with the same amount of the phantom materials in all directions. However, these assumptions do not resemble many clinical procedures such as interstitial and balloon brachytherapy implants in breast, interstitial implant of head and neck, and ${\;}^{125}I$ and ${\;}^{103}Pd$ eye plaque therapy. In these cases, sources are closer to the skin in one direction, while they are surrounded by sufficient scattering materials in other directions. Therefore, the impact of the thickness of missing tissue at one side of the implant on the dose distribution of the treatment volume remained unresolved.

The aim of this work is to investigate the impact of the asymmetric missing tissue on dosimetric characteristics of different brachytherapy sources. This investigation will be performed by determination of the TG‐43–recommended dosimetric parameters of ${\;}^{103}Pd,{\;}^{192}Ir$, and ${\;}^{137}Cs$ brachytherapy sources using MCNP5 Monte Carlo code. The results of this investigation will be compared with the values obtained for the same source models when placed at the center of the phantom with full scattering conditions. One of the commercially available source models from each radioisotope has been selected, assuming that the final results will be applicable for the other source models of the similar isotopes.

## II. MATERIALS AND METHODS

### A. Phantom geometry

**Figure 1:**
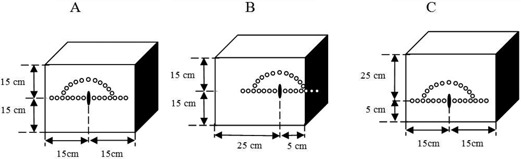
Schematic diagram of the source position at the center (a), at 10 cm shift along the transverse direction of the source (b), and at 10 cm shift along the longitudinal direction of the source (c) within the phantom. These three configurations will be referred to as (15, 15), H (25, 5), and V (25, 5), respectively.

Figure 1 shows the schematic diagram of the phantom geometry used in these investigations. In this phantom geometry, sources are placed either at the center of the phantom (left panel), or shifted along the transverse axis of the source (middle panel), or shifted along the longitudinal axis of the source (right panel). When these source positions are shifted without changing the overall phantom geometry, the thickness of the overlaying phantom material will change. Assuming that the center of the $30\times 30\times 30\;{cm}^{3}$ phantom provides the full scattering condition in all directions, any shift of the source position may invalidate this condition. The differences between the thickness of the overlying tissue (shown as x or y throughout this text) for a given source shift and the thickness of the phantom for a full scattering condition (15 cm) is referred to as missing tissue (i.e., 15‐x or 15‐y) in this work. The impacts of the missing tissue along the transverse and longitudinal axes of the source on its TG‐43 parameters are described in the following sections.

### B. Radioactive sources

#### B.1 ${\;}^{103}Pd$ source

**Figure 2:**
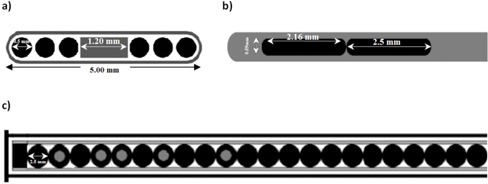
Best Industries, Best Pd‐103, 2335 (a); Varian, VariSource VS2000, HDR Ir‐192 sources (b); and a configuration (c) of several active Cs‐137 LDR sources and several dummy pellets inside the applicator.

The ${\;}^{103}Pd$ source (Model Best2335) from Best Industries (Best Medical International, Inc., Springfield, VA) was used for this investigation. This source design contains a 1.20 mm long and 0.5 mm diameter cylindrical tungsten X‐ray marker at the center of a 5 mm long titanium capsule (0.08 mm thick and 0.8 mm external diameter). There are three spherical polymer resins (each contains 89.73% C, 7.85% H, 1.68% O, and 0.740% N) with a density of $1.00\;g/{cm}^{3}$ at each end of the X‐ray marker, which are coated with ${\;}^{103}Pd$ (see Fig. 2(a)). Per the TG‐43U1 recommendation,^(2)^ the effective active length of this source was found to be 4.55 mm. The TG‐43–recommended dosimetric parameters of this source model have been evaluated and published by two independent investigators^15^, ^16^ and consensus of the results are available in the TG‐43U1 report.^(2)^

#### B.2 ${\;}^{137}Cs$ source

The low‐dose‐rate (LDR) Selectron remote afterloading system distributed by Nucletron (Nucletron B V, Veenedaal, The Netherlands) is utilized in gynecological brachytherapy. Treatments with this unit are normally performed by a combination of several active (A) ${\;}^{137}Cs$ spherical pellets (supplied by Amersham Corporation, now GE Healthcare, Waukesha, WI) and nonactive (N) (dummy) pellets of the same dimensions in an applicator sets.^17^, ^18^ The active cores of these sources are composed of spherical pollucite‐like material, and are 1.5 mm in diameter with a density of $2.9\;g/{cm}^{3}$. These pellets are covered by a 0.5 mm thick stainless steel, with overall diameter of the pellet being 2.5 mm. A mixture of eight active and nonactive pellets (e.g. NANAANAN) produces dose distribution that resembles a ${\;}^{137}Cs$ tube with an active length of 15 mm^17^, ^18^ (see Fig. 2(b)). In this study, the TG‐43 parameters of a combination of eight pellets inside the tandem applicator were obtained using Monte Carlo code (MCNP5) for different diameters of phantoms.

#### B.3 ${\;}^{192}Ir$ source

The VariSource ${\;}^{192}Ir$ (model VS2000; Varian Medical Systems, Palo Alto, CA) was used in this project. The active length of the source is 5 mm with diameter of 0.34 mm (see Fig. 2(c)). The active pellet is embedded in a Ni/Ti (55.6% Ni/44.4% Ti) wire with 0.59 mm diameter (density of $6.5\;g/{cm}^{3}$). For this investigation, the length of wire was extended 5.00 cm from the center of the source during the Monte Carlo simulations, in order to include the impact of the source wire on its dose distribution. The TG‐43 dosimetric characteristics of this source model have been evaluated by several independent investigators.^13^, ^14^

### C. TG‐43 dose calculation formalisms

According to the recommendations of TG‐43 protocol,^1^, ^2^ the absorbed dose rate distribution around a sealed brachytherapy source for line source approximation can be determined using the following formalism:

(1) $$\dot{D}(r,\theta )=\Lambda \;S_{K}\frac{G_{L}(r,\theta )}{G_{L}(r_{0},\theta_{0})}g_{L}(r)F(r,\theta )$$

where Λ is the dose rate constant, $G_{L}(r,\theta )$ is the geometry function, $g_{L}(r)$ is the radial dose function, F(r,θ) is the 2D anisotropy function, and $\left(r_{o}=1\;cm,\theta_{o}=\pi /2\right)$ is the reference point. The above quantities are defined and discussed in detail in TG‐43 reports.^1^, ^2^ The subscript “L” has been added in TG‐43U12 to denote the line source approximation used for the geometry function. $S_{K}$ is the air kerma strength of the brachytherapy source. The dose rate constant was obtained from Eq. (2) as:

(2) $$\Lambda =\frac{\dot{D}\left(1cm,\overset{\pi }{\;}\;/\;\underset{2}{\;}\right)}{S_{k}}$$

The radial dose function, $g_{L}(r)$, describes the attenuation in tissue of the photons emitted from the brachytherapy source. The radial dose function is defined as:

(3) $$g_{L}(r)=\frac{\dot{D}(r,\overset{\pi }{\;}\;/\;\underset{2}{\;})G_{L}(r_{0},\overset{\pi }{\;}\;/\;\underset{2}{\;})}{\dot{D}(r_{0},\overset{\pi }{\;}\;/\;\underset{2}{\;})G_{L}(r,\overset{\pi }{\;}\;/\;\underset{2}{\;})}$$

where $\dot{D}(r,\pi i/2)$ and $\dot{D}(r_{o},\pi /2)$ are the dose rates measured at distances of r and $r_{o}$, respectively, along the transverse axis of the source. The geometry function, $G_{L}(r,\theta )$, takes into account the effect of the activity distribution within the source and the distance between the source and point of interest. The geometry function is defined by the AAPM TG‐43 as:

(4) $$G_{L}(r,\theta )=\{\begin{array}{l} \frac{\beta }{Lr\text{sin}\theta }\;if\;\theta \neq 0^{\circ }\\ {\left(r^{2}‐L^{2}/4\right)}^{‐1}\;if\;\theta =0^{\circ } \end{array}$$

where, β is the angle, in radians, subtended by the point of interest, P(r, θ), to the tips of the active length of a hypothetical line.

2D anisotropy function, F(r, θ) is defined as:

(5) $$F(r,\theta )=\frac{\dot{D}(r,\theta )G_{L}\left(r,\overset{\pi }{\;}\;/\;\underset{2}{\;}\right)}{\dot{D}\left(r,\overset{\pi }{\;}\;/\;\underset{2}{\;}\right)G_{L}(r,\theta )}$$

### D. Monte Carlo calculations

Version 5 of the MCNP MC code (MCNP5) developed by Los Alamos National Laboratory (Los Alamos, NM) was used to perform the simulations in these investigations.^(19)^ There are many different tally types available in MCNP^(20)^ code for scoring diverse physical characteristics. In these investigations, the ^*^F4 tally was utilized to determine the energy flux in $MeV/{cm}^{2}$ that can be converted to absorbed dose by applying a suitable μen/ρ coefficients. In these simulations, the sources were simulated within a cubical water phantom of $30\times 30\times 30\;{cm}^{3}$. In addition, spherical tally cells, with their radii ranging from 0.2 mm to 0.7 mm upon the distances from the source center were utilized in order to minimize the statistical fluctuations of the calculated values. The schematic diagram of our simulation geometries is shown in Fig. 1. All simulations were performed with photon histories of 10^9^ that ensured the standard deviation of simulated data to be less than 0.5% for the tally cell located at distance of 10 cm from the source.

To simulate the air kerma strength ($S_{K}$) of these sources, dry air tally cells were simulated in void phantom for distances ranging from 0.5 to 25 cm. In these simulations, the cutoff energies of 5 keV for ${\;}^{103}Pd$ and 10 keV for ${\;}^{137}Cs$ and ${\;}^{192}Ir$ sources were utilized. Dose rate constant for each source was calculated by dividing the dose rate at reference point (1 cm, π/2) in water phantom by the simulated $S_{K}$.

The radial dose function, g(r), was calculated at radial distances ranging from 0.25 to 10.5 cm. The values of the radial dose function for different source positions were compared with the values obtained from the source position at the center of the 30 × $30\times 30\;{\text{cm}}^{3}$ cubical phantom. The source was moved toward the surface of the phantom along the transverse and longitudinal axes of the source, at distances of 0.5, 1, 2, 4, 5, 6, 8, and 10 cm from the surface of the phantom. The ratio of radial dose functions of ${\;}^{103}Pd,{\;}^{137}Cs$, and ${\;}^{192}Ir$ sources were calculated as a function of sizes of the overlying tissue (x) or missing tissues (15‐x), relative to their radial dose function in full scattering condition (15,15).

(6) $$\text{Perturbation Factor}(r,x)=g_{\left(15,15\right)}^{\left(30‐x,x\right)}(r)=g^{\left(30‐x,x\right)}(r)/g(15,15)(r)$$

for the shift in the direction of the transverse axis of the source, or

(7) $$\text{Perturbation Factor}(r,y)=g_{\left(15,15\right)}^{\left(30‐y,y\right)}(r)=g^{\left(30‐y,y\right)}(r)/g(15,15)(r)$$

for the shift in the direction of the longitudinal axis of the source. The two‐dimensional (2D) anisotropy function, F(r,θ), was calculated at radial distances between 1 to 7 cm and 10° angular intervals between 0° to 90° in both sides of source (toward and away from the missing tissue surface). These calculations were performed for source position at different distances from the phantom surface.

## III. RESULTS & DISCUSSION

### A. Radial dose function

#### A.1 Missing tissue along the transverse axis of the source

Tables 1(a), 1(b), and 1(c) show the radial dose functions of the ${\;}^{103}Pd,{\;}^{137}Cs$, and ${\;}^{192}Ir$ sources, respectively, with their centers 10 cm shifted horizontally (H(25, 5)). These results indicate the values of radial dose function of ${\;}^{103}Pd$ at a distance of 5 cm from the source center on the side away from the missing tissue is larger by up to 7.78% than the side with missing tissue. Differences for ${\;}^{137}Cs$ and ${\;}^{192}Ir$ were 4.89% and 10.10%, respectively. Since dose rates around the brachytherapy sources are directly proportional to the radial dose function, the dose rates

**Table 1:** The percentage difference between the radial dose function of ${\;}^{103}Pd$ (a), ${\;}^{137}Cs$ (b), and ${\;}^{192}Ir$ (c) on the missing tissue side and opposite to the missing tissue side, for H (25, 5) geometry.

(a)			
	*Radial Dose Function for H(25, 5)*		
*Distance, r, (cm)*	*Opposite to the missing tissue side*	*Missing tissue side*	*% Difference*
1.0	1.000	1.000	0.00
1.5	0.770	0.770	0.00
2.0	0.585	0.585	0.00
2.5	0.410	0.410	0.00
3.0	0.318	0.318	0.00
3.5	0.238	0.238	0.00
4.0	0.169	0.167	1.18
4.5	0.127	0.124	2.36
5.0	0.090	0.083	7.78
(b)			
	*Radial Dose Function for H(25, 5)*		
*Distance, r, (cm)*	*Opposite to the missing tissue side*	*Missing tissue side*	*% Difference*
1.0	1.000	1.000	0.00
1.5	0.988	0.988	0.00
2.0	0.981	0.979	0.20
2.5	0.974	0.971	0.31
3.0	0.968	0.962	0.62
3.5	0.961	0.951	1.04
4.0	0.954	0.938	1.68
4.5	0.947	0.922	2.64
5.0	0.940	0.894	4.89
(c)			
	*Radial Dose Function for H(25, 5)*		
*Distance, r, (cm)*	*Opposite to the missing tissue side*	*Missing tissue side*	*% Difference*
1.0	1.000	1.000	0.00
1.5	1.020	1.019	0.10
2.0	1.024	1.021	0.29
2.5	1.022	1.016	0.59
3.0	1.014	1.004	0.99
3.5	1.013	0.994	1.88
4.0	1.024	0.994	2.93
4.5	1.02	0.973	4.61
5.0	1.019	0.916	10.11

on the side away from the missing tissue for ${\;}^{103}Pd,{\;}^{137}Cs$, and ${\;}^{192}Ir$ source would be larger by 7.78%, 4.89%, and 10.10%, respectively.

**Figure 3:**
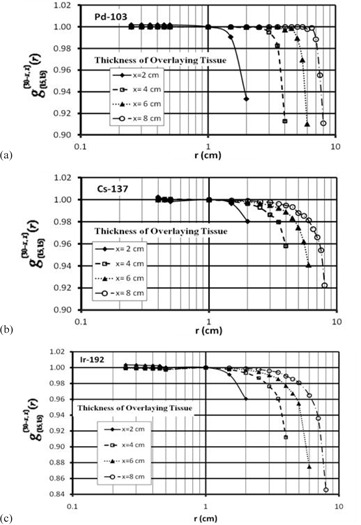
Ratio of radial dose function for different sizes of missing tissues to the radial dose function of for the (15, 15) configuration known as $g_{\left(15,15\right)}^{\left(30-x,x\right)}(r)$ for ${\;}^{103}Pd$ (a), ${\;}^{137}Cs$ (b), and ${\;}^{192}Ir$ (c).

The perturbation factors obtained by (6), (7) indicate that the larger impact of the missing tissue on the radial dose function is associated to a larger thickness of the missing tissue or smaller thickness of the overlaying tissue (see Fig. 3). In addition, the major variations of the radial dose functions occur at the vicinity of the skin or surface of the phantom. These results were expressed in term of a polynomial fit, which could be used as correction factors for dosimetry in the clinical procedures. In these calculations, radial dose function of a source in an implant, with missing tissue, can be calculated from the radial dose function of the source under full scattering condition as:

(8) $$g^{\left(30‐x,x\right)}(r)=\text{Perturbation Factor}(r,x)\times g(15‐15)(r)$$

or

(9) $$g^{\left(30‐y,y\right)}(r)=\text{Perturbation Factor}(r,y)\times g(15‐15)(r)$$

Table 2 shows the coefficients of the polynomial fits to the relative g(r) values of different sources. Table 3 shows a comparison of the results of relative radial dose function of different sources from these investigations at 5 cm distance from the source center for a 10 cm missing tissue or 5 cm thickness of the overlaying phantom materials (i.e., $g_{\left(15,15\right)}^{\left(25,5\right)}(r)$) with the published data by Perez‐Calatayud et al.^(6)^ and Melhus and Rivard^(8)^ for 5 cm radius spherical phantom.

**Table 2:** The coefficients of polynomial fits for ratio of radial dose function for different missing tissue thicknesses to the values of full phantom (i.e., $g_{\left(15,15\right)}^{\left(30-x,x\right)}(r)$) that is expressed in polynomial format as $a_{o}+a_{1}r+a_{2}r^{2}+a_{3}r^{3}+a_{4}r^{4}+a_{5}r^{5}+a_{6}r^{6}$).

	${\;}^{192}Ir$	${\;}^{103}Pd$	${\;}^{137}Cs$
$g_{\left(15,15\right)}^{\left(28+2\right)}(r)$	$a_{o}=1.009E-00$	$a_{o}=1.003E-00$	$a_{o}=1.013E-00$
	$a_{1}=-2.976E-02$	$a_{1}=-4.518E-03$	$a_{1}=‐4.767E‐02$
	$a_{2}=3.632E-02$	$a_{2}=9.935E-03$	$a_{2}=5.188E-02$
	$a_{3}=‐1.476E‐02$	$a_{3}=‐7.712E‐03$	$a_{3}=‐1.811E‐02$
	$a_{4}=‐1.026E‐03$	$a_{4}=‐2.711E‐04$	$a_{4}=0.000E-00$
	$a_{5}=0.000E-00$	$a_{5}=0.000E-00$	$a_{5}=0.000E-00$
	$a_{6}=0.000E-00$	$a_{6}=0.000E‐00$	$a_{6}=0.000E‐00$
$g_{\left(15,15\right)}^{\left(26+4\right)}(r)$	$a_{o}=1.007E‐00$	$a_{o}=9.985E‐01$	$a_{o}=9.888E‐01$
	$a_{1}=‐4.012E‐02$	$a_{1}=7.622E‐03$	$a_{1}=6.047E‐02$
	$a_{2}=6.844E‐02$	$a_{2}=‐1.125E‐02$	$a_{2}=‐1.100E‐01$
	$a_{3}=‐4.830E‐02$	$a_{3}=6.065E‐03$	$a_{3}=9.290E‐02$
	$a_{4}=1.431E‐02$	$a_{4}=‐1.098E‐03$	$a_{4}=‐4.031E‐02$
	$a_{5}=‐1.564E‐03$	$a_{5}=0.000E‐00$	$a_{5}=8.560E‐03$
	$a_{6}=0.000E‐00$	$a_{6}=0.000E‐00$	$a_{6}=‐7.091E‐04$
$g_{\left(15,15\right)}^{\left(25+5\right)}(r)$	$a_{o}=1.005E‐00$	$a_{o}=9.976E‐01$	$a_{o}=9.971E‐01$
	$a_{1}=‐2.917E‐02$	$a_{1}=1.043E‐02$	$a_{1}=1.511E‐02$
	$a_{2}=4.338E‐02$	$a_{2}=‐1.202E‐02$	$a_{2}=‐2.354E‐02$
	$a_{3}=‐2.585E‐02$	$a_{3}=5.117E‐03$	$a_{3}=1.629E‐02$
	$a_{4}=6.332E‐03$	$a_{4}=‐7.043E‐04$	$a_{4}=‐5.977E‐03$
	$a_{5}=‐5.685E‐04$	$a_{5}=0.000E‐00$	$a_{5}=1.081E‐03$
	$a_{6}=0.000E‐00$	$a_{6}=0.000E‐00$	$a_{6}=‐7.777E‐05$
$g_{\left(15,15\right)}^{\left(24+6\right)}(r)$	$a_{o}=9.982E‐01$	$a_{o}=9.978E‐01$	$a_{o}=9.994E‐01$
	$a_{1}=3.291E‐03$	$a_{1}=1.266E‐02$	$a_{1}=3.193E‐02$
	$a_{2}=‐6.178E‐03$	$a_{2}=‐2.361E‐02$	$a_{2}=‐3.879E‐02$
	$a_{3}=5.870E‐03$	$a_{3}=1.897E‐02$	$a_{3}=1.644E‐03$
	$a_{4}=‐2.812E‐03$	$a_{4}=‐7.329E‐03$	$a_{4}=‐4.362E‐04$
	$a_{5}=5.666E‐04$	$a_{5}=1.344E‐03$	$a_{5}=6.241E‐05$
	$a_{6}=‐4.179E‐05$	$a_{6}=‐9.413E‐05$	$a_{6}=‐4.568E‐06$
$g_{\left(15,15\right)}^{\left(22+8\right)}(r)$	$a_{o}=9.973E‐01$	$a_{o}=9.963E‐01$	$a_{o}=9.943E‐01$
	$a_{1}=8.083E‐03$	$a_{1}=1.745E‐02$	$a_{1}=2.180E‐02$
	$a_{2}=‐1.025E‐02$	$a_{2}=‐2.429E‐02$	$a_{2}=‐2.545E‐02$
	$a_{3}=5.884E‐03$	$a_{3}=1.420E‐02$	$a_{3}=1.269E‐02$
	$a_{4}=‐1.737E‐03$	$a_{4}=‐3.955E‐03$	$a_{4}=‐3.099E‐03$
	$a_{5}=2.370E‐04$	$a_{5}=5.202E‐04$	$a_{5}=3.585E‐04$
	$a_{6}=‐1.230E‐05$	$a_{6}=‐2.600E‐05$	$a_{6}=‐1.591E‐05$

**Table 3:** Comparison of the $g_{\left(15,15\right)}^{\left(25,5\right)}(r)$ values calculated at r=5 cm in this study with the published data, $g_{\left(unbounded\right)}^{\left(5\right)}(r)$, for spherical phantoms with 5 cm radius.

	$g_{\left(15,15\right)}^{\left(25,5\right)}(r)$ *(this study)*	$g_{\left(unbounded\right)}^{\left(5\right)}(r)$ *(Perez‐Calatayud et al.* ^(6)^)	$g_{\left(unbounded\right)}^{\left(5\right)}(r)$ *(Melhus and Rivard* ^(8)^)	*% Differences (from this study)*
${\;}^{192}Ir$	0.89	—	0.87	2.3
${\;}^{137}Cs$	0.95	0.93	—	2.2
		—	0.93	2.2
${\;}^{103}Pd$	0.91	0.91	—	0
		—	0.92	1.1

These results indicate an excellent agreement between the results of the present work for ${\;}^{137}Cs,{\;}^{192}Ir$, and ${\;}^{103}Pd$ sources and published data^6^, ^8^ in the direction of the missing tissue. However, unlike the noted published data which show the same effect in all directions (based on a symmetrical phantom geometry), the present data indicate the deviation only in the direction of missing tissue. Therefore, with the published recommendation, one may over‐correct the dose distribution in an asymmetric phantom geometry.

#### A.2 Missing tissue along the longitudinal axis of the source

Tables 4(a), 4(b), and 4(c) show the comparison of g(r) for ${\;}^{137}Cs,{\;}^{192}Ir$, and ${\;}^{103}Pd$, respectively, for 10 cm and 13 cm thick missing tissues (i.e., V(25, 5), V(28, 2)) with the values in a full scattering condition ((15, 15)). These results indicate that the larger missing tissues have larger impact on the radial dose functions of ${\;}^{137}Cs$ and ${\;}^{192}Ir$ than ${\;}^{103}Pd$. For a 13 cm missing tissue, differences of up to 6.61% and 11.57% have been observed at 8 cm depth for ${\;}^{137}Cs$ and ${\;}^{192}Ir$ sources, respectively. However, no significant effect has been observed for ${\;}^{103}Pd$ source. This is due to the fact that the missing tissues have larger effect on the scattering photons in the Compton energy range than low‐energy photons in the photoelectric‐absorption range.

**Table 4:** A comparison between the g(r) values of ${\;}^{137}Cs$ (a), ${\;}^{192}Ir$ (b), and ${\;}^{103}Pd$ (c) sources in V (25, 5), V (28, 2), and (15, 15) configurations.

(a)					
	*g (r)*			*% Difference Between V(15, 15) and V(28, 2)*	*% Difference Between V(15, 15) and V(25, 5)*
*r (cm)*	*V(28, 2)*	*V(25, 5)*	*(15*+*5)*		
1	1.000	1.000	1.000	0.00	0.00
2	0.972	0.976	0.977	0.51	0.10
3	0.977	0.985	0.988	1.11	0.30
4	0.950	0.964	0.970	2.06	0.62
5	0.921	0.941	0.950	3.05	0.96
6	0.906	0.933	0.944	4.03	1.18
7	0.868	0.903	0.917	5.34	1.55
8	0.862	0.904	0.923	6.61	2.10
9	0.833	0.881	0.901	7.55	2.27
10	0.788	0.842	0.867	9.11	2.97
(b)					
	*g (r)*			*% Difference Between V(15, 15) and V(28, 2)*	*% Difference Between V(15, 15) and V(25, 5)*
*r (cm)*	*V(28, 2)*	*V(25, 5)*	*(15*+*5)*		
1	1.000	1.000	1.000	0.00	0.00
2	1.016	1.023	1.026	0.97	0.29
3	0.990	1.007	1.014	2.37	0.70
4	0.984	1.012	1.024	3.91	1.19
5	0.962	1.003	1.021	5.78	1.79
6	0.969	1.024	1.049	7.63	2.44
7	0.903	0.968	0.999	9.61	3.20
8	0.841	0.914	0.951	11.57	4.05
9	0.815	0.898	0.940	13.30	4.68
10	0.768	0.857	0.904	15.04	5.48
(c)					
	*g (r)*			*% Difference Between V(15, 15) and V(28, 2)*	*% Difference Between V(15, 15) and V(25, 5)*
*r (cm)*	*V(28, 2)*	*V(25, 5)*	*(15*+*5)*		
1	1	1	1	0.00	0.00
2	0.564	0.565	0.565	0.18	0.00
3	0.3086	0.31	0.31	0.45	0.00
4	0.1686	0.17	0.17	0.82	0.00
5	0.0898	0.091	0.091	1.32	0.00
6	0.0549	0.056	0.056	1.96	0.00
7	0.0272	0.028	0.028	2.86	0.00
8	0.0154	0.016	0.016	3.75	0.00
9	0.011	0.0116	0.0117	5.98	0.86
10	0.0041	0.0043	0.0043	4.65	0.00

### B. Anisotropy functions

#### B.1 Missing tissue along the transverse axis of the source

**Figure 4:**
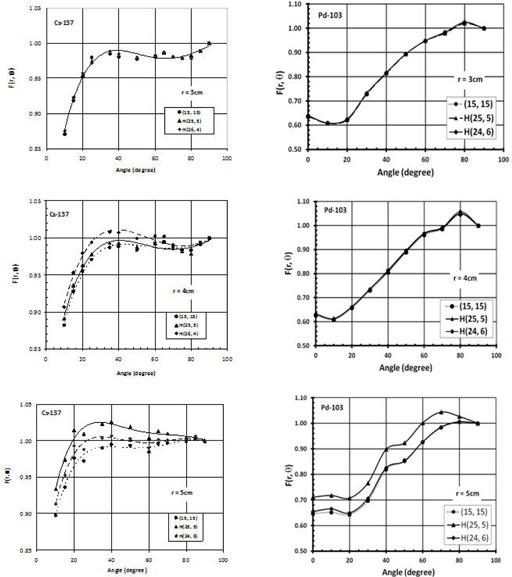
A comparison of the anisotropy function of ${\;}^{137}Cs$ and ${\;}^{103}Pd$ sources at radial distances of 3 cm (top panel), 4 cm (middle panel), and 5 cm (lower panel) with their centers shifted by 10 cm and 11 cm along the transverse axis of the source (i.e., H (25, 5) and H (26, 4)) relative to the values with the source centers at the phantom center (i.e., (15, 15). The ${\;}^{137}Cs$ source was composed of six active (AAAAAA) pellets.

The values of anisotropy function for the sources at different positions inside the phantom were evaluated in this study. Figure 4 shows a comparison between the anisotropy function of the ${\;}^{137}Cs$ (left panel) and ${\;}^{103}Pd$ (right panel) sources at distances of r=3,4, and 5 cm, with 9 cm and 10 cm source shift along the transverse axis (H(24, 6) and H(25, 5)) relative to full scattering conditions (15, 15). The ${\;}^{137}Cs$ source was composed of six active (AAAAAA) pellets. These results indicate that the impact of missing tissue is more significant at larger distances with smaller overlaying tissue. The increase of the anisotropy functions is attributed to the decrease of the dose rate along the transverse bisector of the source. At a 25° angle and 5 cm radial distance, the anisotropy function of ${\;}^{137}Cs$ and ${\;}^{103}Pd$ changes by approximately 4% and 10%, respectively, for the 10 cm source shift (H(25, 5)).

#### B.2 Missing tissue along the longitudinal axis of the source

**Figure 5:**
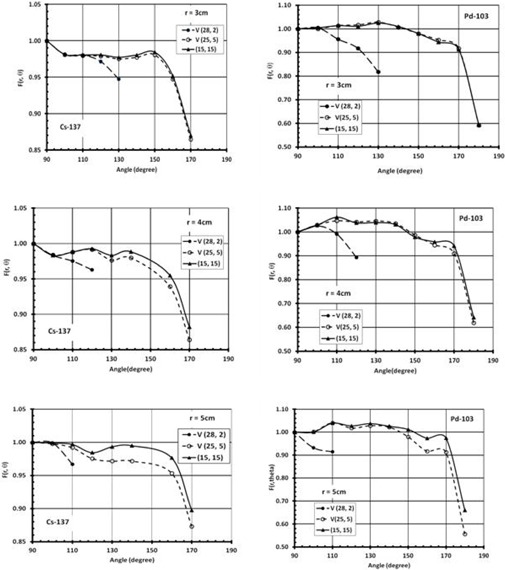
The anisotropy functions of ${\;}^{103}Pd$ and ${\;}^{137}Cs$ sources at radial distances of 3 cm (top panel), 4 cm (middle panel), and 5 cm (lower panel) with their centers shifted by 13 cm along the longitudinal axis of the source (i.e., V (28, 2)) relative to the values with the source centers at the phantom center (i.e., (15, 15). The ${\;}^{137}Cs$ source was composed of six active (AAAAAA) pellets.

Figure 5 shows a comparison between F(r, θ), of the ${\;}^{137}Cs$ (left panel) and ${\;}^{103}Pd$ (right panel) for V(28, 2) and (15, 15), at radial distances of r=3,4, and 5 cm. These results indicate that the values of F(r, θ) decreases significantly for the points towards the missing tissue. However, there was no effect from the missing tissue on the data points located away from the missing tissue. In addition, the range of angles and radial distances that are affected by the missing tissue depend on the missing tissue configuration. For V (28, 2) configuration, the point (r=3 and θ>130°) is located outside of the phantom, while it is inside the phantom for (15, 15). However, the anisotropy function of ${\;}^{137}Cs$ and ${\;}^{103}Pd$ for r=3 and θ≤130° show difference of up to 6% and 18%, respectively.

### C. Dose rate constant (Λ)

Table 5 shows the comparison of the dose rate constant, A, values of ${\;}^{137}Cs,{\;}^{192}Ir$, and ${\;}^{103}Pd$ brachytherapy sources for different missing tissue configurations. These results indicate that the missing tissue does not have any significant effect (i.e., greater than 5%) on the dose rate constant of these sources, except for very large missing tissue (i.e., when reference points fall at the close vicinity of the phantom surface) where the source is located at close vicinity of the surface.

**Table 5:** Dose rate constants of ${\;}^{137}Cs,{\;}^{192}Ir$, and ${\;}^{103}Pd$ brachytherapy sources using different thicknesses of missing tissues along the transverse and longitudinal directions of the sources.

*Source*	*Phantom Geometry*	*Dose Rate Constant* $\left(cGy.h^{-1}.U^{-1}\right)$	*% Differences from Full Scattering Condition*
${\;}^{137}Cs$	(15, 15)	1.093	—
	H(25, 5)	1.090	0.3
	V(25, 5)	1.088	0.5
	H(28, 2)	1.091	0.2
	V(28, 2)	1.055	3.6
${\;}^{192}Ir$	(15, 15)	1.109	—
	H(25, 5)	1.120	1.0
	V(25, 5)	1.108	0.1
	H(28, 2)	1.108	0.1
	V(28, 2)	1.105	0.4
${\;}^{103}Pd$	(15, 15)	0.650	—
	H(25, 5)	0.650	0
	V(25, 5)	0.650	0
	H(28, 2)	0.650	0
	V(28, 2)	0.650	0

## IV. CONCLUSIONS

TG‐43 dosimetric parameters of the brachytherapy sources are normally designed for a phantom geometry that provides full scattering conditions. Several investigators have examined the variation of these parameters for different size phantoms, with the source being located at the center of a symmetrical phantom. In the present investigation, the effects of missing phantom material (or the thickness of the overlaying tissues) on TG‐43 dosimetric parameters of ${\;}^{137}Cs,{\;}^{192}Ir$ and ${\;}^{103}Pd$ brachytherapy sources were investigated in an asymmetric phantom. These geometric arrangements more closely represent real clinical setups, such as interstitial breast implants. The results of these investigations show that the effects of missing tissues are more pronounced on the radial dose function and anisotropy functions of the sources. Unlike the previously published data^6^, ^8^ which were based on the symmetric phantom geometry around the source, the present data indicate that the effect of the missing tissue is only visible to the dose distribution that is closer to the missing tissue, and may not have a significant impact on the dose distribution away from the missing tissue. Therefore, care should be taken in implementation of these corrections. Moreover, the anisotropy functions are also affected by the missing tissue.

The dose rate constants of the brachytherapy sources do not change significantly, unless the reference point is located near the phantom surface.

In summary, the missing tissue will only affect the radial dose function and 2D anisotropy functions of the brachytherapy sources in the direction of the missing tissue (no effect in the other directions). This effect is more pronounced on radial dose function when the source is parallel to the skin, while it is significant on 2D anisotropy function when the source is perpendicular to the skin. Dose rate constant is not affected significantly by the missing tissue, unless the source is parallel to the skin and it is at the close vicinity of the skin.

## ACKNOWLEDGMENTS

The authors of this manuscript would like to present their appreciation to Drs. Courtney Knaup and Shahid Awan for their valuable comments and suggestions during the preparation of the manuscript.
